# Extracellular HSP60 triggers tissue regeneration and wound healing by regulating inflammation and cell proliferation

**DOI:** 10.1038/npjregenmed.2016.13

**Published:** 2016-10-27

**Authors:** Wuhong Pei, Katsuya Tanaka, Sunny C Huang, Lisha Xu, Baoying Liu, Jason Sinclair, Jennifer Idol, Gaurav K Varshney, Haigen Huang, Shuo Lin, Robert B Nussenblatt, Ryoichi Mori, Shawn M Burgess

**Affiliations:** 1 Functional and Translation Genomics Branch, National Human Genome Research Institute, Bethesda, MD, USA; 2 Department of Plastic and Reconstructive Surgery, School of Medicine and Graduate School of Biomedical Sciences, Nagasaki University, Nagasaki, Japan; 3 Laboratory of Immunology, National Eye Institute, Bethesda, MD, USA; 4 Department of Molecular, Cell, and Developmental Biology, University of California Los Angeles, Los Angeles, CA, USA; 5 Laboratory of Chemical Genomics, School of Chemical Biology and Biotechnology, Shenzhen Graduate School of Peking University, Shenzhen, China; 6 Department of Pathology, School of Medicine and Graduate School of Biomedical Sciences, Nagasaki University, Nagasaki, Japan

## Abstract

After injury, zebrafish can restore many tissues that do not regenerate well in mammals, making it a useful vertebrate model for studying regenerative biology. We performed a systematic screen to identify genes essential for hair cell regeneration in zebrafish, and found that the heat shock protein Hspd1 (Hsp60) has a critical role in the regeneration of hair cells and amputated caudal fins. We showed HSP60-injected extracellularly promoted cell proliferation and regeneration in both hair cells and caudal fins. We showed that *hspd1* mutant was deficient in leukocyte infiltration at the site of injury. Topical application of HSP60 in a diabetic mouse skin wound model dramatically accelerated wound healing compared with controls. Stimulation of human peripheral blood mononuclear cells with HSP60 triggered a specific induction of M2 phase CD163-positive monocytes. Our results demonstrate that the normally intracellular chaperonin HSP60 has an extracellular signalling function in injury inflammation and tissue regeneration, likely through promoting the M2 phase for macrophages.

## Introduction

Hearing loss, affecting millions of people worldwide, is primarily caused by the death of mechanosensory hair cells in the inner ear. In contrast to humans and all other mammals, many non-mammalian vertebrates, including zebrafish, can replace the dead hair cells and fully recover hearing loss. All vertebrates possess some ability to regenerate tissue after traumatic injury. Although mammalian tissue regeneration can be relatively limited and is inhibited by fibrotic scarring, many other vertebrates can regrow neural tissue, organs or even entire limbs after damage that are structurally or functionally indistinguishable from the originals.^1,2^ These models for regeneration offer valuable information on identifying the common elements that are required for wound healing or regeneration, which will ultimately inform studies on human regenerative medicine.^3^

Injury-induced tissue regeneration in vertebrates comprises several distinct phases, including an inflammatory response, wound closure, cell proliferation and structural restoration. Each of the processes is governed by a precise molecular programming that guides specific cell behaviour. Although numerous genes and signalling cascades have been shown to be involved in these processes,^3–5^ there are still many critical questions remaining about the molecular mechanisms behind the regeneration of different tissues, such as what are the critical signals released from the injury site and how these signal regulate regenerative growth.

Zebrafish has become a popular vertebrate model for studying regeneration. Many tissues, particularly ones that do not typically regenerate in mammalian systems, such as the heart, brain or limb (fin),^6,7^ can be studied. In this study, we identified Hspd1/Hsp60 (*no blastema, nbl*)^8^ from a targeted screen for mutants deficient in hair cell regeneration. Hspd1 is a critical factor in injury-induced regeneration of hair cells as well as caudal fin regrowth after amputation. It has previously been shown that Hspd1 can specifically induce an inflammatory response through several different receptors including TLR2, TLR4 and CD36.^9–11^ We show that Hspd1 acts as an extracellular signal released from the injury site, acting both as a chemoattractant for leukocytes and as an inflammation-resolving signal that promotes cell division and regeneration in the surrounding tissues. We also show that providing ectopic HSP60 to a skin wound in a diabetic mouse model is sufficient to restore normal wound healing, suggesting an important therapeutic use for HSP60 in diabetic patients.

## Results

### *hspd1* is necessary for hair cell and fin regeneration

**Figure 1:**
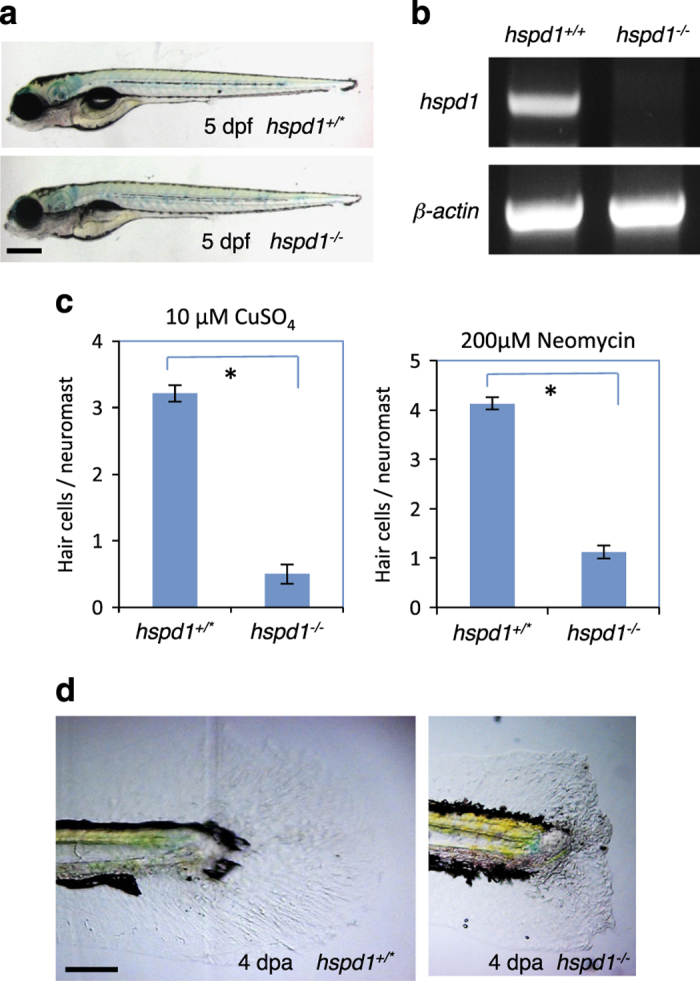
*hspd1* mutants display deficient regeneration of lateral line hair cells and caudal fins. (**a**) The morphology of *hspd1* mutants looks normal except for the lack of an inflated swim bladder at 5 dpf. *hspd1*^+/*^ are wild-type or heterozygous fish. *hspd1*^*−/−*^ are confirmed homozygous mutant fish. (**b**) RT-PCR analysis of *hspd1* mRNA expression. The retroviral DNA is inserted in the first intron of the *hspd1* gene, and the first exon is noncoding. The primers used for *hspd1* knockdown analysis bind to the exon 3 and exon 6. β-actin is used as an internal reference. (**c**) Hair cell regeneration analysis using CuSO_4_ or neomycin to ablate hair cells. Concentrations are as labelled. The reduction is significant for both treatments (*n*=10, *P*<0.001 for both copper and neomycin). Asterisks in the graphs indicate a significant difference between the control and mutant embryos. (**d**) Caudal fin regeneration is deficient in *hspd1*^*−/−*^ mutants. The end of the tail was removed at 3 dpf, and regeneration evaluated at 4 dpa, and then phenotype was correlated to genotype. Defects in fin regeneration were observed in 1/21 of *hspd1*^+/*^ embryos, and 14/18 of *hspd1*^*−/−*^ embryos. Bars = 500 μm in **a** and 200 μm in **d**. dpa, day post amputation; dpf, day post-fertilisation; RT-PCR, reverse transcriptase PCR.

To understand the mechanisms of hair cell regeneration, we performed a large-scale reverse genetics screen to identify genes having an essential role in the regeneration of hair cells in the zebrafish lateral line (Pei and Burgess, unpublished data), targeting genes identified by transcriptional profiling in Liang *et al.*^12^ We identified Hspd1/Heat Shock Protein 60 as a key factor in hair cell regeneration. The *hspd1*^*la026911*^ mutation was generated by a retroviral DNA insertion in the first intron of the *hspd1* gene.^13^ The homozygous mutants had a normal appearance, but failed to inflate their swim bladder (Figure 1a) and survived only for the first 2 weeks of embryo development. Reverse transcriptase PCR (RT-PCR) analysis showed no expression of either *hspd1* wild-type (WT) or truncated mRNA in the homozygous mutants (Figure 1b). *hspd1* mutants displayed normal neuromast patterning and hair cell development (Supplementary Figure S1), but had severely impaired hair cell regeneration after hair cell ablation by the ototoxic drugs copper or neomycin (Figure 1c). A temperature-sensitive allele of *hspd1* (*nbl*) was reported to be deficient in adult caudal fin regeneration;^8^ therefore, we examined whether the *hspd1*^*la026911*^ allele had a role in larval caudal fin regeneration. We performed larva fin amputation and found that *hspd1*-homozygous mutants failed to regenerate the amputated caudal fin similar to the *nbl* adults (Figure 1d). Taken together, these data demonstrate that Hspd1 is required for both hair cell and fin regeneration and potentially other forms of wound healing in zebrafish.

**Figure 2:**
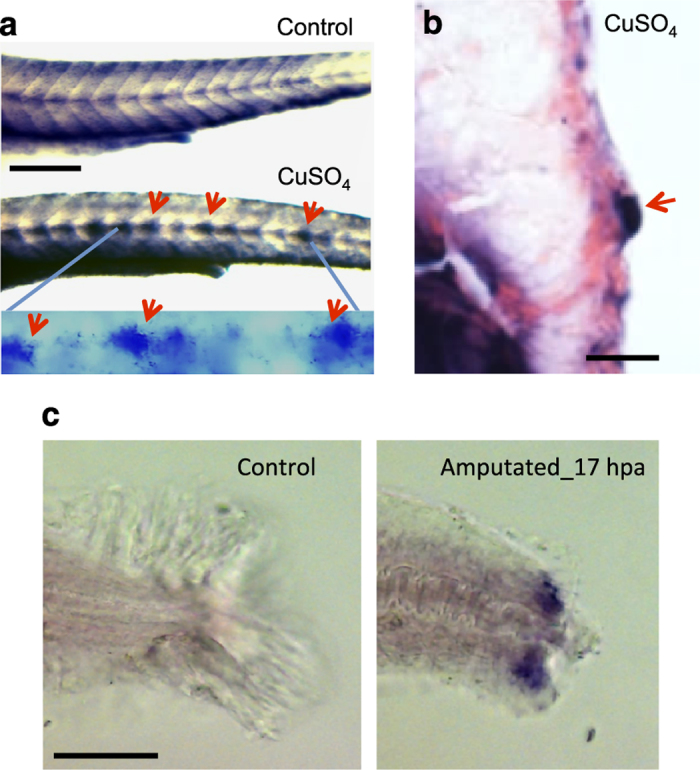
*hspd1* expression is induced after injury. (**a**) *hspd1* expression is induced in lateral line neuromasts by CuSO_4_-mediated hair cell ablation. Pictures are of embryos collected 5 h post-copper or control treatment, the time point with the largest expression differences. Arrows indicate the induced expression in lateral line neuromasts. The bottom panel is a higher magnification to more clearly show the neuromast-specific expression only seen in a CuSO_4_-treated embryo. (**b**) Histological sectioning shows the induced *hspd1* expression in a cross-section of a neuromast localised in the trunk. (**c**) *hspd1* expression is induced by caudal fin amputation. Pictures are of embryos collected at 17 h post amputation (or an undissected control), which was the peak of *hspd1* expression. Bars = 200 μm in **a** and **c**, 20 μm in **b**.

Whole-mount *in situ* analysis (WISH) showed that *hspd1* was not enriched in expression in the lateral line neuromasts or caudal fins during embryo development (Supplementary Figure S2A). To examine *hspd1*’s expression during wound healing, we performed a time-course analysis of *hspd1* expression during hair cell and caudal fin regeneration. Five hours after hair cell ablation induced by copper sulfate exposure, a strong induction of *hspd1* expression was observed in a solid circular pattern in the neuromasts. This neuromast expression was further verified by histological sectioning (Figure 2a,b). As there are no new hair cells formed by 5 h post ablation, the solid circular expression indicates that *hspd1* gene expression was in the supporting cells of the neuromast and possibly other nearby surrounding cells. During caudal fin regeneration, amputation-induced *hspd1* expression became detectable at 3 h post amputation, reached its peak at 17 h and then receded (Figure 2c and Supplementary Figure S2B). Histological sectioning analysis revealed that *hspd1* was expressed predominantly in the cells situated in the mid-line region of the caudal fin (Supplementary Figure S2C), an area where the blastema typically forms.^14^

### *hspd1* is linked to immune responses during injury

**Figure 3:**
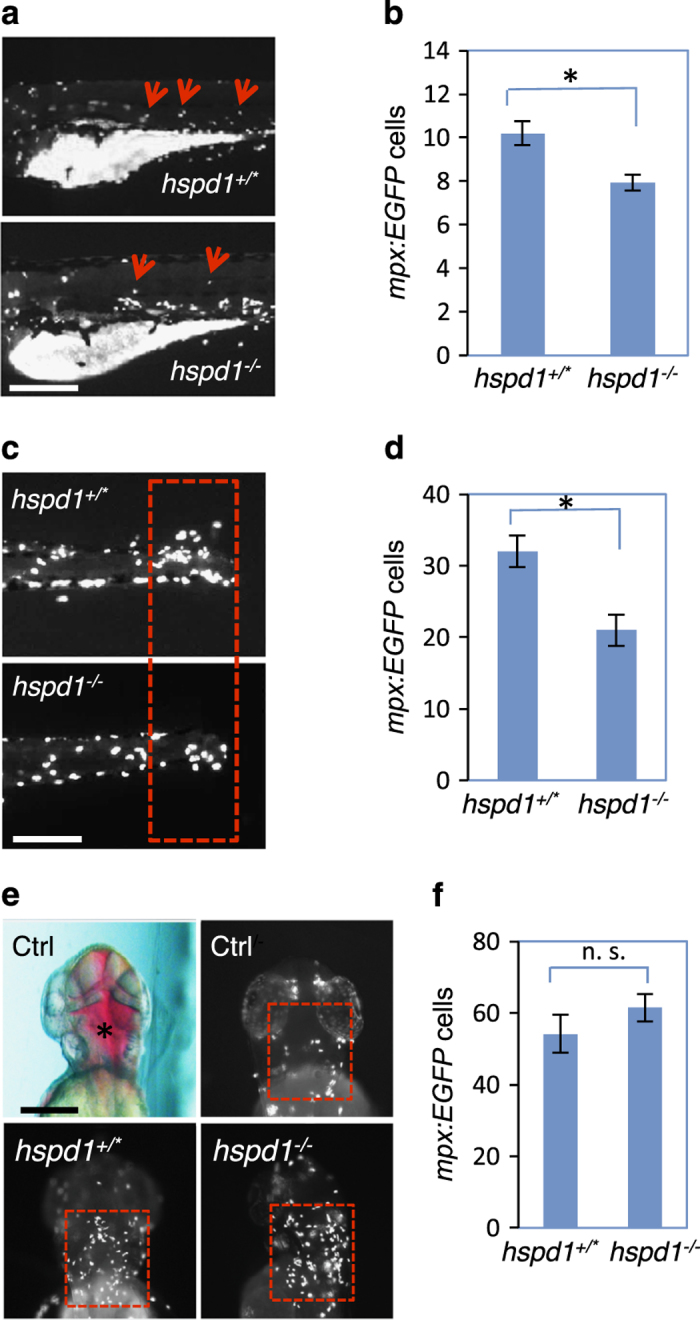
*hspd1* mutants have impaired neutrophil migration towards the injury site. (**a**) Neutrophil migration is triggered by hair cell ablation. Neutrophils were labelled by Tg(*mpx*:*EGFP*) and pictures were taken after 1 h of CuSO_4_ treatment. Arrows point to neutrophil accumulation in lateral line neuromast areas. (**b**) Quantification of the neutrophils that migrated to the regions surrounding the neuromasts; scoring was done before the genotypes were known. The reduction is significant (*n*=12, *P*=0.001). (**c**) Neutrophil migration triggered by caudal fin amputation. Pictures were taken at 17 h post amputation, when the difference in the number of migrated neutrophils between control and mutant embryos was greatest. (**d**) Quantification of the neutrophils migrated to the amputated fin at 17 h post amputation; scoring was done before the genotypes were known. Red boxes demarcate the areas used for quantification. The reduction is significant (*n*=10, *P*<0.001). (**e**) Neutrophil migration triggered by injection of LPS. The top panels show the control embryos injected with phenol red buffer and BSA. The red box in the top right panel shows a low number of *mpx*:EGFP cells accumulated in the BSA injection area. The bottom panels show control and *hspd1* mutant embryos injected with 150 pg LPS. Red dotted boxes show that LPS injection strongly attracts neutrophils into the injection area. (**f**) Quantification of neutrophils migrated to the LPS injection site in *hspd1* control and mutant embryos. Red dotted boxes demarcate the areas used for quantification. There is no significant difference between mutant and control in neutrophil’s ability to migrate (*n*=10, *P*=0.291). Asterisks indicate a significant difference. Bars = 200 μm. BSA, bovine serum albumin; LPS, lipidpolysaccharide.

Immune cell activation and migration are one of the earliest responses triggered by tissue injury. To study whether the *hspd1* mutation affects immune cell migration, we crossed *hspd1* mutants into a Tg(*mpx*:*EGFP*) transgenic background, which has green fluorescent protein (GFP) that marks neutrophils specifically.^15^ Neutrophil development and patterning in *hspd1* mutants was indistinguishable from control siblings (Supplementary Figure S3A). Hair cell ablation caused neutrophils to migrate towards damaged neuromasts, as previously reported.^16^ We found that the number of neutrophils migrating to hair cell-ablated neuromasts was significantly reduced in *hspd1* mutants (Figure 3a,b). Similarly, a significantly reduced number of neutrophils was observed in amputated caudal fins of *hspd1* mutants at 17 h post amputation (Figure 3c,d), at the time when *hspd1* expression normally reached its highest level (Supplementary Figure S2B). Both results point to an association between neutrophil migration and *hspd1* expression level, consistent with the result from published *in vitro* studies.^17^

To investigate whether the *hspd1* mutation affects the ability of neutrophils to migrate, lipidpolysaccharide (LPS), a widely used inflammation inducer and powerful leukocyte attractant, was injected into the brain ventricle of control and *hspd1* mutant embryos. We observed rapid migration of *mpx*:*EGFP*-positive neutrophil towards the injection area in both control and *hspd1* mutants, with no significant difference detected between two groups (Figure 3e,f). Together, these data demonstrate that *hspd1* mutants possess a normal number of neutrophils fully competent to migrate to a wound site; however, their migration towards injury sites was reduced in the absence of *hspd1* expression, suggesting that expression of *hspd1* in the wound area was participating in attracting cells of the innate immune response.

We also crossed *hspd1* mutants into a Tg(*mpeg:EGFP*) transgenic background, in which GFP is specifically expressed in macrophages.^18^ Macrophage development and patterning in *hspd1* mutants was indistinguishable from control siblings (Supplementary Figure S3B). Macrophage migration was slightly reduced between the control siblings and the mutant embryos at 17 h post caudal fin amputation, but the reduction did not reach statistical significance (*P*=0.11; Supplementary Figure S3C,D). These data suggest that macrophage migration is more weakly drawn to the injury site by *hspd1* than by neutrophils.

### Extracellular HSP60 protein acts as a chemoattractant for leukocytes

**Figure 4:**
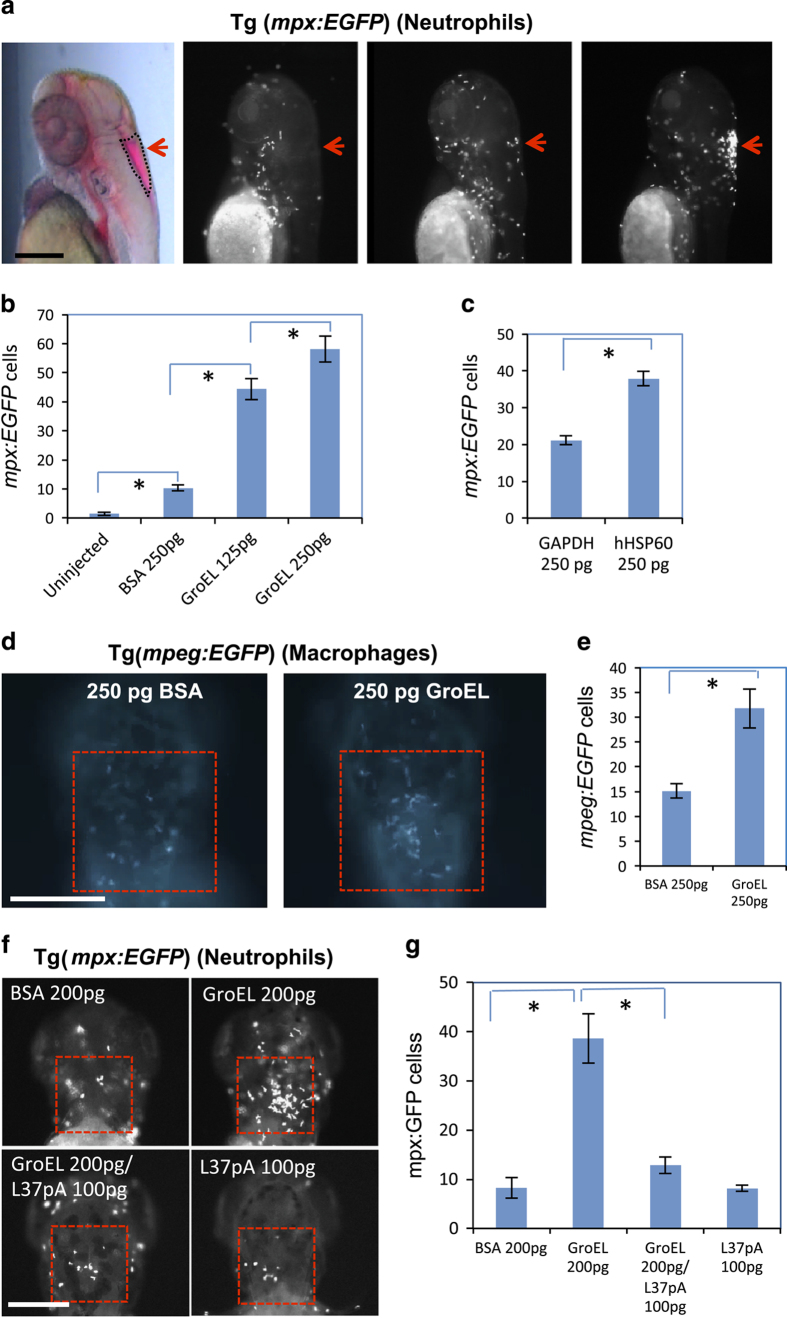
Exogenous HSP60 attracts both neutrophils and macrophages. (**a**) GroEL injected into the brain ventricle attracted *mpx*-positive neutrophils. Pink region outlined in first panel shows tracer dye in the zebrafish brain ventricle. Red arrows in the other three panels indicate injection site in the uninjected, control protein injected, or GroEL/human HSP60 injected embryos. (**b**) Quantification of neutrophil infiltration stimulated by GroEL. There is a significant increase in neutrophil numbers between the indicated two groups (*n*=10, *P*<0.05 for all comparisons). (**c**) Quantification of neutrophil infiltration stimulated by human HSP60 injected into the brain ventricle. Human HSP60 attracts significantly more neutrophils than human GAPDH (*n*=12, *P*<0.001). (**d**) GroEL injected into the brain ventricle attracted *mpeg*-postive macrophages. (**e**) Quantification of macrophage infiltration stimulated by GroEL. There is a significant increase in Tg(*mpeg*:*EGFP*) cells upon GroEL injection (*n*=8, *P*=0.003). (**f**) Inhibitory peptide L-37pA blocks GroEL’s neutrophil chemoattraction. Injection for each condition was into the brain ventricle. GroEL elicited a strong chemoattractive response, but the inhibitor of CD36, L37pA, prevented infiltration. Pictures were taken 3 h post injection into the brain ventricle and neutrophils were labelled by Tg(*mpx*:EGFP). Red boxes demarcate the areas in which neutrophils were quantified. (**g**) Quantification of the experiments in **f**. The increase in Tg(*mpx:EGFP*) cells is significant upon GroEL injection (*n*=10, *P*<0.001), but not significant upon GroEL/L37pA co-injection (*n*=10, *P*=0.109) or L37pA injection alone (*n*=10, *P*=0.962). Asterisks in the graphs indicate a significant difference. Bars = 200 μm. BSA, bovine serum albumin.

The major cellular function of Hspd1 is to act as a chaperonin in the cytosol and mitochondrial matrix;^19^ however, it is difficult to envision a model where Hspd1 acts as a local chemoattractant while simultaneously being sequestered intracellularly. We therefore formulated a hypothesis where Hspd1 was released into the extracellular space, either via ‘leaking’ caused by cell damage or apoptosis, or via active secretion by the cells, which has been previously shown in cell culture.^20^ To test whether extracellular Hspd1 could be responsible for stimulating leukocyte migration, we injected recombinant, *Escherichia coli* GroEL (51% identical and 72% similar to zebrafish Hspd1) into the brain ventricle, which caused a rapid accumulation of *mpx*:*EGFP*-positive neutrophils at the injection site (Figure 4a,b), at a level comparable to that of the LPS injections (Supplementary Figure S4A,B). In contrast, injection of bovine serum albumin (BSA) as a control protein caused very low levels of neutrophil accumulation that served as a baseline for neutrophil attraction from injection-related injury. GroEL’s chemoattractant activity was further verified by using two additional transgenic lines: Tg(*lyz:DsRED*) that also labels neutrophils^21^ and Tg(*mpeg:EGFP*) that specifically labels macrophages.^18^ Injection of GroEL into the brain ventricle strongly attracted *lyz:DsRED-*positive cells (Supplementary Figure S4C,D) and *mpeg*:*EGFP*-positive cells (Figure 4d,e).

The role of HSP60 as a chemoattractant was further verified by the injection of recombinant human HSP60 (87% identical to zebrafish Hspd1 and 94% similar) and recombinant human GAPDH. Recombinant human HSP60 produced a similar effect as *E. coli* GroEL in attracting *mpx:EGFP* cells (Figure 4c), although with a weaker activity. The difference in the activity in attracting neutrophils between human HSP60 and *E. coli* GroEL could be attributed to their protein stability, or their specific interactions with the innate immunity system.^22^

GroEL is known to activate the Toll-Like Receptors 2 and 4 (TLR2 and TLR4)^23^ as well as scavenger receptor CD36.^9,24^ To demonstrate that the chemoattraction was a result of signalling through HSP60, we used the peptide inhibitor L-37pA that specifically competes with HSP60 for CD36 receptor activation.^9^ When co-injected with GroEL into the brain ventricle, L-37pA nearly eliminated leukocyte migration to the injection site (Figure 4f,g), suggesting that the chemoattractive inflammatory response to HSP60 is primarily through binding the CD36 receptor. L-37pA had no effect on LPS chemoattraction (Supplementary Figure S4G,H), which signals through TLR4, supporting L-37pA as a specific inhibitor of GroEL chemoattraction.

**Figure 5:**
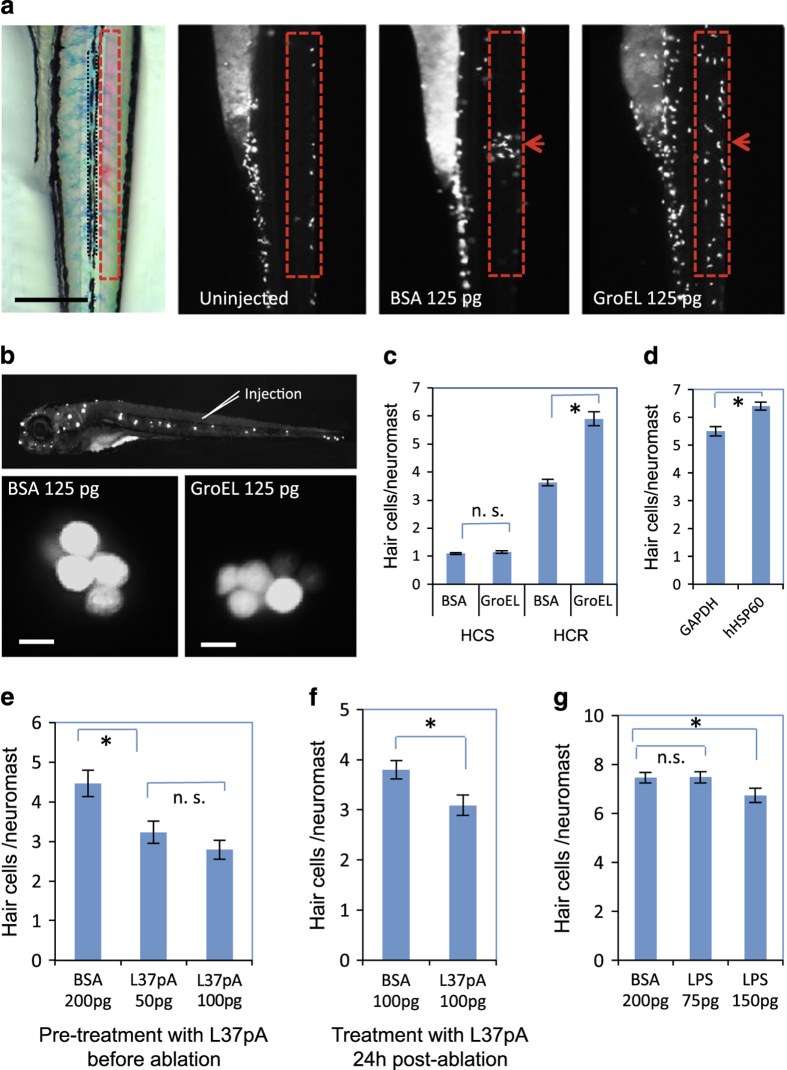
Exogenous HSP60 promotes hair cell regeneration and signalling inhibitor L-37pA inhibits regeneration. (**a**) GroEL injected into the trunk attracted *mpx*-positive neutrophils similar to brain ventricle injections. The areas framed with red dotted lines in the left panels indicate the areas of injection (the panel on the left) or quantification (three panels on the right). The red colour shown within the dotted lines of the first panel is from phenol red in the injection buffer. BSA protein is used as a control for GroEL protein and also a control for assessing the needle-induced injury. Red arrows point to the sites of injection. (**b**) GroEL injected into the trunk promotes hair cell regeneration. The top panel shows a 7 dpf zebrafish larvae stained with Yopro-1, with each fluorescent dot indicating a hair cell containing neuromast. The injection site is pointed. The bottom panels show representative images of hair cells in a neuromast for each condition. (**c**) Quantification of hair cell sensitivity to copper (left) and rate of regeneration (right). GroEL does not provide protection from cell death (*n*=16, *P*=0.471), but does significantly boost regeneration rates (*n*=15, *P*<0.001). (**d**) Quantification of hair cell regeneration stimulated by human HSP60. Human HSP60 injected into the trunk promotes hair cell regeneration significantly (*n*=34 for GAPDH, *n*=36 for human HSP60, *P*<0.001). (**e**) L-37pA injected into the trunk before hair cell ablation inhibits hair cell regeneration (*n*=8, *P*=0.002). Injection of 50 or 100 pg of L-37pA inhibits regeneration to a similar degree (*n*=8, *P*=0.176). (**f**) L-37pA injected into the trunk 1 day after hair cell ablation inhibits hair cell regeneration (*n*=13, *P*=0.01). For all experiments, hair cells were ablated in WT embryos at 5 dpf, and then hair cell regeneration evaluated at 7 dpf. (**g**) LPS injected into the trunk does not promote hair cell regeneration. Injection of 75 pg of LPS does not affect hair cell regeneration (*n*=8, *P*=0.938), whereas injection of 150 pg of LPS caused a slight but significant inhibition of regeneration (*n*=8, *P*=0.023). Asterisks in the graphs indicate a significant difference between the indicated two groups. Bars = 200 μm in (**a**), 10 μm in (**b**). dpf, days post-fertilisation. BSA, bovine serum albumin; HCR, hair cell regeneration; HCS, hair cell sensitivity; LPS, lipidpolysaccharide; WT, wild type.

Similar leukocyte mobilisation was seen when GroEL was injected into the middle of the larval trunk. The presence of GroEL in the dorsal trunk of *mpx*:*EGFP* or *lyz:DsRED* embryos caused the normally ventrally localised leukocytes to migrate dorsally, spreading across the entire trunk area (Figure 5a, data not shown). These data demonstrate that extracellular Hspd1 acts as a mobilising chemoattractant for the immune system in zebrafish, similar to reports in mammals.^25^

### Extracellular HSP60 stimulates tissue regeneration

Although leukocyte migration to the injury site is triggered immediately after injury, the resulting inflammatory response can either have a positive or a negative impact on regeneration depending on the local context of the injury.^26–29^ To study the effect of extracellular HSP60 on hair cell regeneration, GroEL or human HSP60 was injected into the trunk at a position above the end of yolk extension, so that extracellular HSP60 and leukocyte activation were in close proximity to the regenerating hair cells of the lateral line. Unlike reports for the protective effects of extracellular Hsp70 on hair cells of the mouse utricle,^30^ GroEL injection did not protect hair cells from copper-induced apoptosis. However, it did stimulate hair cell regeneration significantly compared with BSA-injected control group (Figure 5b,c). Similar to GroEL, recombinant human HSP60 also promoted hair cell regeneration (Figure 5d). On the other hand, CD36 inhibitor L37pA caused a significant reduction in the level of hair cell regeneration (Figure 5e). All these data demonstrate the importance of extracellular HSP60 signalling to the regeneration response. A higher dose of L-37pA (100 pg) did not cause a further inhibition to a level as severe as that in *hspd1* mutants, suggesting that the extracellular Hspd1 may only partially contribute to stimulating regeneration or the inhibition by the peptide is insufficient to block all signalling.

Because hair cell ablation caused a local induction of *hspd1* expression, it raised the possibility that the locally induced Hspd1 is the protein that can be secreted into extracellular matrix over time to further facilitate the regeneration. To address this possibility, we injected L-37pA at 1-day post-hair cell ablation when the impact of locally released extracellular Hspd1 from apoptotic hair cells would have been removed. Injection of L-37pA at 1 day post-hair cell ablation still caused a significant reduction in hair cell regeneration (Figure 5f), suggesting that a continuous supply of secreted Hspd1 was necessary for regeneration. Moreover, we observed that injected GroEL was not sufficient to rescue the defective hair cell regeneration in *hspd1* mutant larvae (data not shown). These data demonstrate that a constant supply of extracellular Hspd1 is needed during the hair cell regeneration process.

Although LPS resulted in a strong, dose-dependent neutrophil attraction to the injection site identical to GroEL (Supplementary Figure S4A,E,F), LPS injection into the trunk did not promote hair cell regeneration, and a higher dose (150 pg) of LPS actually caused a slight inhibition of hair cell regeneration in larvae (Figure 5g). Therefore, although both GroEL and LPS equally attracted leukocytes, it was specifically GroEL-induced inflammation that was beneficial to hair cell regeneration.

**Figure 6:**
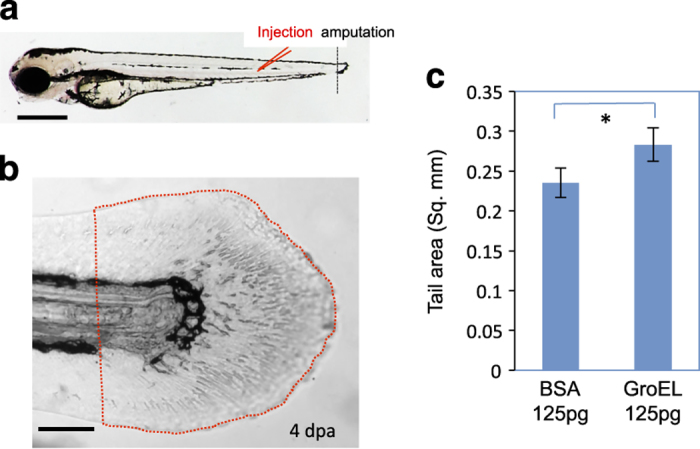
Extracellular HSP60 promotes caudal fin regeneration. (**a**) Schematic of injection site and amputation site. (**b**) Caudal fin area measured in the injected embryos at 4 dpa. Quantified areas are framed with dotted red lines, starting from the anterior end of the ventral pigmentation break. (**c**) Quantification of caudal fin regeneration in GroEL- and BSA-injected embryos. A significant increase (indicated by an asterisk) in the fin area is detected in GroEL-injected embryos (*n*=10, *P*=0.003). Bars = 500 μm in **a**, 100 μm in **b**. BSA, bovine serum albumin; dpa, day post amputation.

We confirmed the role of extracellular Hspd1 in caudal fin regeneration by injecting GroEL into the distal trunk of WT embryos, performing caudal fin amputation and then analysing fin regeneration (Figure 6a). We found that the caudal fins regenerated in GroEL-injected embryos were significantly larger than those of BSA-injected embryos (Figure 6b,c), indicating that extracellular HSP60 also promotes caudal fin regeneration. To test whether the increased regeneration induced by injected GroEL is due to a tropic effect, we injected GroEL to unamputated fins and found no significant difference on caudal fin development (Supplementary Figure S5). These data, taken together with our observations of the normal hair cell development and defective hair cell regeneration in *hspd1* mutant, suggest that injury provides a sensitised background that can exaggerate HSP60’s function in regenerative cell proliferation.

Extracellular Hspd1 is known to bind to cell surface TLR 2 and 4 and CD36, whereas LPS binds to TLR4 and CD36.^9,23,31–34^ Our data showed that exogenous Hspd1/GroEL, but not LPS, promotes tissue regeneration, suggesting that the signalling through TLR2 could be beneficial to tissue regeneration. To test this possibility, TLR2 ligands Pam3CSK4 (for TLR1/2) and FSL-1 (for TLR2/6) were injected to evaluate their effect on inflammation and hair cell regeneration. A significant accumulation of *mpx*-positive neutrophils was detected when either of the two ligands was injected into the brain ventricle (Supplementary Figure S6A), although the accumulation levels were much lower than those for GroEL or LPS (Figure 4 and Supplementary Figure S4). No detectable effect was observed on hair cell regeneration when injected into the trunk (Supplementary Figure S6B). Owing to the relatively low activities of these purified TLR ligands, it remains unclear whether tissue regeneration can be enhanced by activation of TLR2 only, TLR2 and another receptor(s) or if it is occurring through some other mechanism.

### Extracellular HSP60 triggers cell proliferation

**Figure 7:**
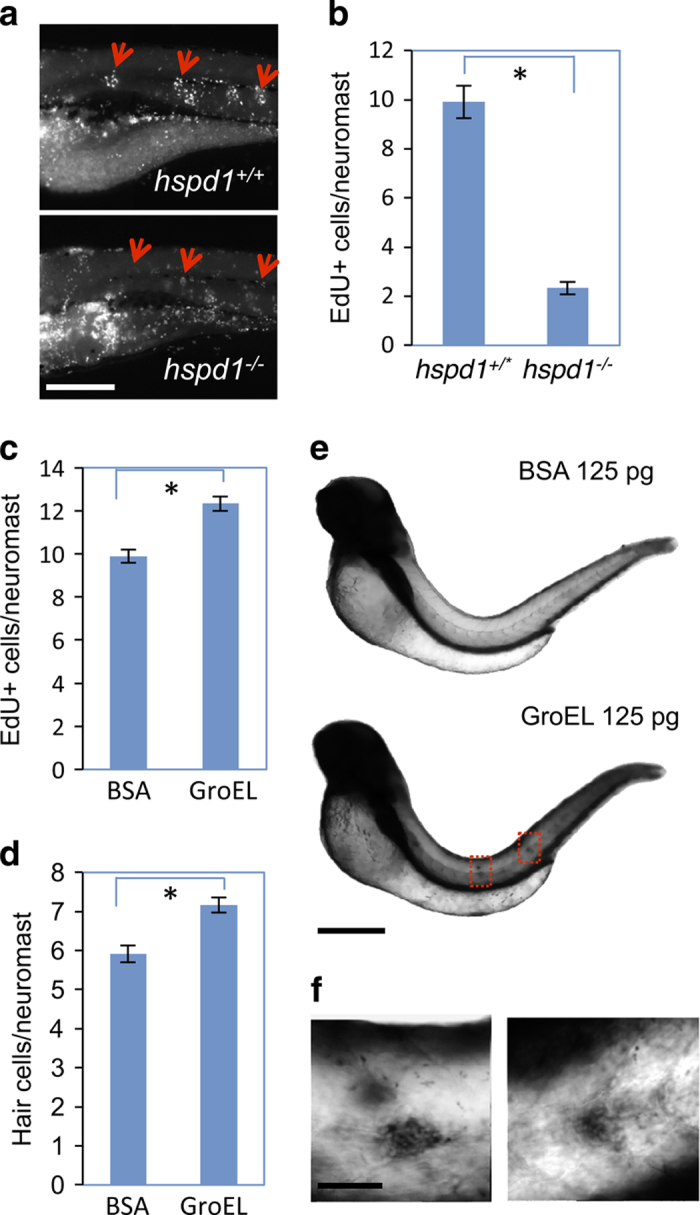
*hspd1* is necessary for cell proliferation, and extracellular HSP60 stimulates intracellular *hspd1* expression. (**a**) Impaired proliferation of supporting cell in *hspd1* mutants, assayed by EdU labelling analysis. Five-day-old control and *hspd1*^*−/−*^ mutant embryos were used for hair cell ablation, EdU labelling and then quantified for cell proliferation. Pictures shown are examples from 24 h post hair cell ablation. Arrows point to the proliferating supporting cells in the lateral line neuromasts. (**b**) Quantification of the EdU signal. Quantification performed before genotype was determined (*n*=12, *P*<0.001). (**c**) Exogenous GroEL promotes cell proliferation during regeneration. WT embryos at 5 dpf were injected with GroEL, hair cells were ablated and dividing cells labelled with EdU. Data shown were quantified at 24 h post ablation (*n*=14, *P*<0.001). (**d**) Exogenous GroEL promotes supernumerary hair cells. WT embryos at 2 dpf were injected with GroEL into the trunk. Hair cells were counted at 5 dpf by Yopro-1 staining (*n*=16, *P*=0.006). (**e**) GroEL injected in the trunk induces *hspd1* expression in the trunk and lateral line neuromasts as revealed by whole-mount *in situ* hybridisation. WT embryos at 2 dpf were used for 125 pg of GroEL or BSA injection and for analysing *hspd1* expression at different time points after the injection by whole-mount *in situ* hybridiation. Pictures shown are representative examples from 7 h post injection. GroEL-injected embryos showed darker staining in the trunk and lateral line neuromasts. Two red boxes frame the enriched expression of *hspd1* in two neuromasts. (**f**) The magnified images of the two boxed areas of GroEL-injected embryos, revealing the induced *hspd1* expression specifically in the neuromasts. Asterisks in **b**–**d** indicate a significant difference between the two groups. Bars = 200 μm in **a**, 500 μm in **e** and 50 μm in **f**. dpf, days post-fertilisation. BSA, bovine serum albumin; WT, wild type.

Inducing cell proliferation is a critical process during tissue regeneration. To investigate whether Hspd1 has a role in initiating cell proliferation, we first analysed the proliferation of supporting cells after hair cell ablation in control and *hspd1* mutants. We performed hair cell ablation for 5-day-old control and *hspd1* mutant embryos incubated with 5-ethynyl-2′-deoxyuridine (EdU), and then analysed supporting cell proliferation. EdU labelling demonstrated a significant reduction of proliferating supporting cells in *hspd1* mutants (Figure 7a,b). We tested whether extracellular HSP60 could directly stimulate cell division by injecting GroEL into the trunk, ablating hair cells 3 h later followed by incubation with EdU. A significant increase in EdU-positive cells was observed in GroEL-injected embryos (Figure 7c). Furthermore, we injected GroEL into the trunk at the onset of lateral line development (2 days post fertilisation) and evaluated GroEL’s effect on hair cell development at 5 days post fertilisation in the absence of hair cell ablation. A significantly increased number of hair cells were found in GroEL-injected embryos when compared with BSA-injected embryos (Figure 7d), indicating that GroEL promoted hair cell proliferation even in the absence of actual injury. These EdU-labelling experiments indicated that extracellular HSP60 can stimulate cell proliferation directly.

We tested whether extracellular HSP60 could act in a paracrine manner to activate its own expression. We injected GroEL into the trunk and measured *hspd1* expression by whole-mount *in situ* in the embryos collected at different time points after injection. A significant induction of intracellular *hspd1* expression was detected in the embryos collected at 7 h post-GroEL injection. The induced *hspd1* expression was detectable in the trunk, with stronger expression in lateral line neuromasts (Figure 7e). High-magnification imaging of *hspd1* in the neuromasts (Figure 7f) revealed a solid circular expression pattern that presumably contains both hair cells and supporting cells. It is unclear which receptors are acting to induce local *hspd1* expression in these cells.

### HSP60 accelerates wound healing in diabetic mice

**Figure 8:**
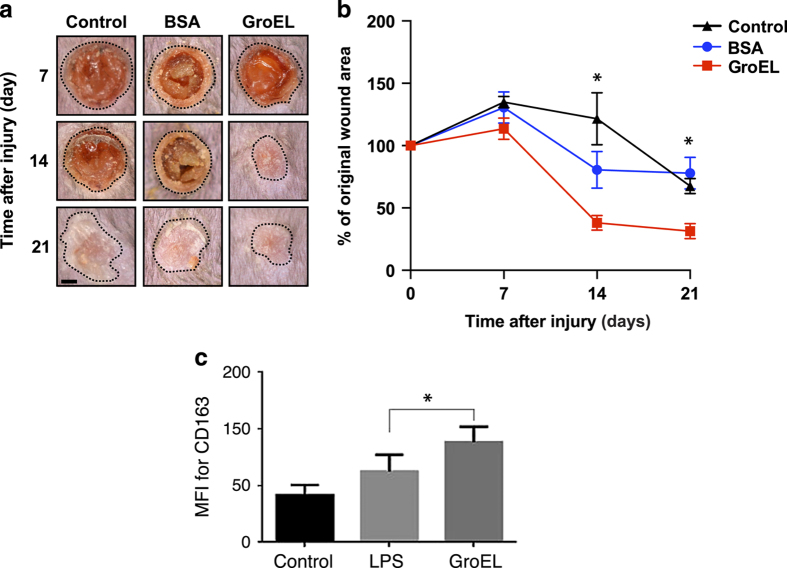
Ectopic application of HSP60 stimulates wound healing in diabetic mice and stimulates M2 macrophages in human peripheral blood cells. (**a**) Representative images of skin puncture wounds of *db*/*db* mice on the back at 7, 14 and 21 days after the initial injury. Dotted black lines demarcate the wound opening. Bar = 1 mm. (**b**) Quantification of wound healing in the untreated control, BSA-treated or GroEL-treated wounds over a 21-day test period. Wound size is expressed as a percentage of the initial wound area. The number of wounds and mice used for the treatment and quantification: 12 wounds from 6 mice for the untreated control and 9 wounds from 9 mice for the BS or GroEL-treated. The difference is significant at 14 and 21 days between untreated control and GroEL-treated, or between BSA-treated and GroEL-treated mice (*P*<0.05 for all). The difference is not significant for all the time points between untreated and BSA treatment. The statistical analysis was carried out by one-way analysis of variance. (**c**) Human peripheral blood mononuclear cells stimulated with either LPS or GroEL for 24 h are measured for expression of the M2 marker CD163. GroEL significantly increased M2 phase macrophages over LPS (*P*=0.006). Asterisks indicate a significant difference. BSA, bovine serum albumin; LPS, lipidpolysaccharide.

Considering the role of extracellular HSP60/GroEL in regulating inflammation and promoting tissue regeneration, we tested its effect on skin wound healing in a diabetic mouse model, *Lepr*^*db*^*/ Lepr*^*db*^, which has been characterised with an abnormal immune response and impaired wound healing.^35–38^ Two 4-millimetre diameter wounds were made on the dorsal skin of homozygous *Lepr*^*db*^*/ Lepr*^*db*^ mice. We then performed an ectopic application of 100 μg of BSA or GroEL suspended in 30% Pluronic F-127 gel. We found that BSA-treated wounds showed no clear signs of healing during the 21-day period we monitored. However, GroEL-treated wounds showed a dramatic and significant improvement in wound healing over the same time period (Figure 8a,b). At 21 days post puncture, GroEL-treated wounds were largely healed, whereas BSA-treated wounds still showed no significant signs of closing. To rule out the possibility that BSA may have a negative effect on wound healing, we compared the healing of BSA-treated and -untreated control wounds, and found no significant difference between them.

### HSP60 induces M2-like monocytes

To further investigate the differences in the inflammatory responses mediated by Hspd1/GroEL or LPS, we stimulated human peripheral blood mononuclear cells (PBMCs) with GroEL or LPS and measured CD163 levels, a scavenger receptor that is usually highly expressed by M2 macrophages.^39^ We found that the intermediate CD14^++^CD16^+^ cells after GroEL treatment exhibited a higher level of CD163 expression than the cells from LPS treatment (Figure 8c), suggesting that GroEL has a role in inducing the differentiation of intermediate monocytes to M2 macrophages, biasing the inflammation response towards resolution and regeneration.

## Discussion

On the basis of the data from this study, we propose a working model illustrating the mechanism of Hspd1-mediated tissue regeneration (Supplementary Figure S7). Injury-induced cell death, from hair cell ablation or caudal fin amputation, causes a release of mitochondrial Hspd1 into the extracellular matrix. The extracellular HSP60 acts both as an immunostimulant to attract leukocytes into the injury site and as a paracrine signal that induces intracellular *hspd1* expression in the neighbouring cells. This increased intracellular expression likely results in an increase in secreted HSP60, which continues to modulate the local inflammatory response. The macrophages drawn to the injury by HSP60 are stimulated to polarise towards the M2 state, encouraging tissue regeneration.^40^ Coordinating leukocyte chemoattraction, inflammation resolution and cell proliferation by extracellular HSP60 constitutes the specific mechanism behind the *hspd1*-mediated tissue regeneration and demonstrates that HSP60 has a ‘moonlighting’ function as an extracellular signalling molecule during wound healing.

This study provides evidence for the importance of *hspd1* in the regeneration of neuromast hair cells and caudal fins in zebrafish larvae. Consistent with our data, other studies also have reported the requirement of heat shock proteins in vertebrate tissue regeneration. For example, the Hsp60 V324E mutant, also known as *no blastema*, contains a missense mutation in *hspd1* and displays deficiency in the regeneration of caudal fin, heart and retina in adult zebrafish.^8,41^ In contrast to the early lethality observed in our *hspd1*^*la026911*^ allele and in a mouse *hspd1* inactivating gene-trap mutant,^42^ *nbl* mutants can survive to adulthood at a permissive temperature of 25 °C and only displays defective tissue regeneration at the non-permissive temperature of 33 °C. Studies also have implicated HSP70 in muscle and liver regeneration in mouse models,^43,44^ and HSP90 in wound healing in mouse.^45^ It will be interesting to understand whether these heat shock proteins regulate tissue regeneration through a shared mechanism, with extracellular signalling being a key component, or whether their functions activate independent pathways.

Our data indicate that injury-released extracellular HSP60 is an essential trigger for the regeneration of hair cells and caudal fins. We propose that injury-related cell death releases mitochondrial or cytoplasmic HSP60 into the extracellular matrix where it acts as the initial source of extracellular HSP60. Pre-injury injection of L-37pA blocks hair cell regeneration, suggesting that the initial extracellular signalling function of HSP60 is an essential component of the regeneration trigger (Figure 5e). Pre-injury addition of HSP60 boosts the endogenous signalling and thus promotes an increase in regeneration (Figures 5a–d and 6). However, we noticed that an exogenous supply of GroEL failed to restore the hair cell regeneration deficiency in *hspd1* mutants (data not shown), suggesting that a continuous supply of HSP60 is required in the injured area during the regeneration process. This *hspd1* expression is induced in a paracrine manner by the presence of extracellular HSP60, and in the *hspd1*^*la026911*^ mutants this induction is not possible resulting in a failure to regenerate. As regeneration can be reduced by L-37pA even 24 h after the initial injury (Figure 5f), these data suggest that the increased intracellular HSP60 is subsequently secreted into the extracellular space to stimulate local regeneration for a sustained period. Consistent with our observations that exogenous HSP60 promotes the regeneration of hair cells and caudal fins, studies in mouse have shown that topical application of HSP90 enhances wound healing,^45^ and a supply of extracellular HSP70 restores the inflammation deficiency required for muscle regeneration after injury.^43^ Like HSP90 and HSP70, HSP60 does not possess a secretion signal peptide; its secretion to the extracellular space (in the absence of cell damage) appears to be through an exosome pathway.^20,46^

Extracellular HSP60 attracts leukocytes and triggers inflammatory responses through interacting with cell surface receptors. Additional work will be required to assess the specific functions of TLR2, TLR4 and/or CD36 activation in this context. Regardless of the specifics of TLR signalling or which set of cytokines are being induced downstream of HSP60 signalling, an immune reaction has been shown to be necessary to trigger proper regeneration.^27^ Our data show the requirement of leukocyte infiltration during regeneration, consistent with the findings from other studies.^27,47–51^ In addition, transcriptional analysis has revealed that several immune response genes are associated with the regeneration of the heart and retina in zebrafish.^41^ Altogether, these findings present strong evidence that inflammation is a necessary predecessor to tissue regeneration in zebrafish.

Several lines of evidence point to a role for HSP60 in cell proliferation during wound healing. (i) Lack of Hsp60 in the *hspd1* mutants impaired regenerative cell proliferation (Figure 7a,b) even though basic developmental growth was not affected. (ii) An exogenous supply of HSP60 promoted regenerative cell proliferation regardless of the presence or absence of injury (Figure 5a–d, Figure 7b,c). (iii) Injection of HSP60 leads to an increased number of hair cells (Figure 7d) in the absence of injury. How much of the cell proliferation activity can be attributed to extracellular HSP60 versus its intracellular chaperonin function remains unclear. Our data show that extracellular HSP60 induces intracellular *hspd1* expression (Figure 7e). Some studies have reported that intracellular HSP60 regulates the proliferation of stem cells and cancer cells.^52–54^ However, others show that exogenous HSP60 in cell culture promotes cell proliferation.^23,55^ It is worth noting that extracellular HSP60 is being investigated as a potential target for the diagnosis and treatment of some types of cancers because of its high level of expression and its extracellular location.^56^ There is some evidence that TLR4 can act as an intracellular receptor,^57^ adding the possibility that an increase in cytoplasmic HSP60 could directly trigger TLR4 signalling intracellularly. It requires further studies to parse the molecular mechanisms of extracellular HSP60 versus the necessity for intracellular HSP60 on cell proliferation.

Most dramatic was the effect that HSP60 protein had on wound healing in diabetic (*db*/*db*) mice. In general, puncture wounds in *db*/*db* mice do not heal properly over the course of many weeks, a problem similar to that of human diabetic patients. Topical application of HSP60 results in a near-complete wound healing of *db*/*db* mice within 21 days (Figure 8a,b). There are two lines of evidence from the literature that suggest that an absence of HSP60 could be linked to the deficit in wound healing in diabetics. First, leptin signalling has been shown to positively regulate *hspd1* expression,^58,59^ suggesting that *db*/*db* mice might have a wound-healing deficit because of a reduction in *hspd1* expression, thereby resulting in regeneration defects similar to those in the *hspd1*^*la026911*^ mutant zebrafish. Second, recent evidence shows that neutrophils isolated from diabetic mice or humans are more primed to form neutrophil extracellular traps that inhibit wound healing.^60^ We argue that the reduced levels of extracellular HSP60 protein in the diabetic patients means that the inflammatory response is not efficiently being resolved into the wound-healing state, instead of remaining in the microbial defense state. The addition of topical HSP60 could reduce the neutrophil extracellular trap response and independently trigger more *hspd1* expression, allowing the wound to heal on a normal time course.

In summary, this study demonstrates that HSPD1/HSP60 is an essential factor in regulating inflammatory response and cell proliferation during tissue regeneration, providing new evidence for the importance of an extracellular function of HSP60 in triggering tissue regeneration. This pro-regeneration function is true across a variety of different tissues and is conserved across species as diverse as fish and humans, suggesting that it is a fundamental wound-healing signal for innate immunity.

## Materials and methods

### Biological materials and zebrafish transgenic lines

Biological materials used in this study are as follows: LPS (Sigma, Cat#: L4130, St Louis, MO, USA); recombinant GroEL (MyBioSource, Cat# MBS650332, San Diego, CA, USA); recombinant human GAPDH (MyBioSource, Cat# 203254); recombinant human HSP60 (Abcam, Cat# ab78792, Cambridge, MA, USA); L-37pA, a gift from Dr Alexander Bocharov;^9^ TLR2 ligands Pam3CSK4 and FSL-1 (InvivoGen, Cat# tlrl-pms, Cat# tlrl-fsl, San Diego, CA, USA); and Click-It EdU Alexa Fluor 555 imaging Kit (Life Science, Cat# C10338, Waltham, MA, USA). Zebrafish transgenic lines used are as follows: Tg(*mpx*:EGFP)^i114^;^15^ Tg(*lyz*:*DsRed*);^61^ Tg(*mpeg*:*EGFP*);^18^ and *nbl*^*la026911*^.^13^

### Animal husbandry

Zebrafish husbandry and embryo staging were performed according to Kimmel *et al.*^62^ and in compliance with the National Institutes of Health (NIH) guidelines for animal handling and research. All experiments were approved by the NHGRI Animal care and Use Committee (protocol G-01-3). To study *hspd1* mutant morphology and mRNA expression and regeneration phenotypes, individual embryos from a single pair of heterozygous carriers of *hspd1*^*la026911*^ were used for the analysis and were then genotyped with primers hspd1-WT (5′- AGAACACATGTGCGTCGAGT-3′), hspd1-q (5′- CCTGCCTGTTTGAGCTCACTGATT-3′) and 3LTR222 (5′- ACCAATCAGTTCGCTTCTCGCTTC-3′; WT allele, 230 bp; mutant allele, 315 bp) to evaluate the genotype–phenotype correlation. For RT-PCR analysis of *hspd1* expression in mutant embryos, total RNA was extracted by Trizol (Invitrogen, Carlsbad, CA, USA) from individual embryos at 3 days post-fertilisation (dpf) obtained from a heterozygote incross, and cDNA was synthesised using the SuperScript first-strand synthesis system (Invitrogen). β-actin was used as an internal reference.

### Hair cell quantification

Hair cell staining and counting were as described.^63^ For analysis of hair cell development, WT or mutant embryos at 5 dpf were placed in a cell strainer (BD Falcon, San Jose, CA, USA) and stained with 2 μmol/l YoPro-1 (Life Science) for 5–15 min and then lateral line neuromast hair cells were counted using fluorescent imaging (inverted Zeiss Axiophot, ×10 magnification, Bethesda, MD, USA). For hair cell sensitivity analysis, WT or mutant embryos at 5 dpf were treated with the ototoxic drugs copper sulfate (Sigma) at 10 μmol/l for 30 min or neomycin (Sigma) at 200 μmol/l for 30 min, and then were immediately stained with YoPro-1 and used for hair cell counting. For hair cell regeneration analysis, WT or mutant embryos at 5 dpf were treated with the ototoxic drugs copper sulfate at 10 μmol/l for 2 h or neomycin at 200 μmol/l for 1 h, allowed to recover for 2 days and then regenerated hair cells were counted at posterior lateral line positions of P1, P2, P4 and P5.^64^ For each of the above experiments, ~10 embryos were used for the analysis and repeated three times. Hair cells from four neuromasts in each embryo were counted. The average number of hair cells and the s.e.m. were presented in the graphs.

### Quantification of caudal fin development and regeneration

WT and/or mutant embryos at 3 dpf were anaesthetised and used for caudal fin amputation. The amputation plane was posterior to the blood circulation at the rear end of the ventral pigmentation gap in the caudal fin; therefore, the anterior end of the ventral pigment break can serve as a landmark for the analysis of the regeneration of multiple fin tissues. Fin regeneration was analysed at 7 dpf by imaging the regenerated fins, outlining, measuring and calculating the total area of growth individually and then genotyping the embryos by PCR when needed. For caudal fin development analysis, WT embryos were used for BSA or GroEL injection at the posterior trunk at 3 dpf, and for caudal fin area measurement at 7 dpf. Fin area was measured using Image J (NIH, Bethesda, MD, USA). Quantification data were obtained from analysing ~10 embryos per data point, except otherwise indicated, and repeated three times. Graphs show the mean and s.e.m.

### WISH and histological sectioning

WISH was carried out as previously described.^65^ For WISH on embryos older than 24 h post fertilisation, N-phenylthiourea (Sigma) was used to suppress pigmentation. For hair cell ablation-induced *hspd1* expression, WT embryos at 5 dpf were incubated with or without 10 μM copper sulfate for 2 h and then fixed at different time points for WISH. For fin amputation–induced *hspd1* expression, we amputated caudal fins from WT embryos at 3 dpf and then fixed at different time points for WISH. For histological sectioning after WISH, single embryos were embedded in paraffin, transversely sectioned at a thickness of 5 μ and then stained with fast nuclear red (HistoServ, Germantown, MD, USA).

### Injury-induced leukocyte migration analysis

For injury-induced leukocyte migration analysis in *hspd1* mutants, the embryos were obtained from crossing a *hspd1*^*la026911*^ heterozygote with a *hspd1*^*la026911*^/Tg(*mpx*:EGFP) or a *hspd1*^*la026911*^/Tg(*mpeg*:GFP) heterozygote. The 5-day-old embryos from this cross were sorted, and GFP-positive embryos were used to analyse leukocyte migration triggered by hair cell ablation or fin amputation. The number of leukocytes in a consistently defined region around four different neuromasts per fish was counted. Counts of *mpx:EGFP* cells were obtained with the Zeiss Axio Observer A1 microscope. Counts of *mpeg:EGFP* cells were obtained with Zeiss 510 NLO meta confocal microscope (Bethesda, MD, USA) and the Imaris image analysis software (Concord, MA, USA). Counts shown in graphs were obtained by analysing ~10 embryos per group (repeated three times). The embryos were then genotyped for genotype–phenotype correlation analysis.

### Microinjection of immunoreactive ligands

The injection buffer was made with 1× PBS containing 1 mg/ml phenol red (Sigma). Injection solutions were freshly made before each injection by diluting the stock solution with injection buffer to the desired concentration. Microinjection was performed using a World Precision Pump injection system with anaesthetised embryos placed in a soft agarose bed. For BSA, GroEL, human GAPDH, human HSP60, L-37pA and LPS injections for leukocyte migration and hair cell regeneration, WT and/or mutant embryos at 5 dpf were used. Injection volume for brain ventricle injections was ≈1 nl, using embryos oriented with a dorsal view. Injection volume for the dorsal vessel of the trunk was ≈ 0.5 nl, using embryos oriented with a lateral view. BSA was used as a control for GroEL, and GAPDH was used as a control for human HSP60. The effect of injections on leukocyte migration was analysed 3 h post injection. For hair cell regeneration analysis, injected embryos at 3 h post injection were used for hair cell ablation and then analysed for regeneration at 48 h post ablation. For the effects of GroEL or BSA injections on caudal fin regeneration, WT embryos were injected in the dorsal vessels of the posterior trunk at 3 days post fertilisation, and caudal fins were amputated 3 h post injection and then analysed for fin regeneration 4 days post amputation. For the effect of GroEL and BSA on caudal fin development, a similar procedure was followed, except no fin amputation was performed.

### *Lepr*^*db*^/*Lepr*^*db*^ mouse skin wound-healing analysis

Heterozygotic *Lepr*^*db*^/^+^ mice were incrossed to obtain homozygotic *Lepr*^*db*^/*Lepr*^*db*^ mice.^35–37^ Homozygous *Lepr*^*db*^/*Lepr*^*db*^ mice at 8–12 weeks were used for skin puncture and wound-healing analysis as previously described.^66^ In brief, two full-thickness excisional wounds of 4-mm diameter were made to the shaved dorsal midline skin of each mouse. Each wound was ectopically applied with 100 μg of GroEL or BSA that was pre-mixed with 50 μl of 30% Pluronic F-127 gel (Sigma, Cat #P2443), or used for untreated control. To avoid the anterior–posterior location effects, GroEL and BSA were applied to wounds at different anterior–posterior positions for different experiments. The wound areas were imaged and measured at 0, 7, 14 and 21 days after the skin puncture. Graph shows the quantification data from analysing the wound healing of 15 homozygous *Lepr*^*db*^/ *Lepr*^*db*^ mice.

### Human PBMC stimulation

The study was approved by the Institutional Review Board of the NIH and conformed to the tenets of the Declaration of Helsinki. Human blood samples from four healthy individuals were obtained from the NIH blood bank. Human PBMCs were isolated from the blood of healthy donors using a Ficoll gradient centrifugation protocol. PBMCs were then treated with buffer vehicle, LPS (5 μg/ml) or GroEL (5 μg/ml) for 24 h, followed by anti-CD14-APC, anti-CD16-FITC and anti-CD163-PE staining (BD Biosciences, San Jose, CA, USA). Fluorescent cells were acquired on a flow cytometer (BD FACSCalibur, San Jose, CA, USA) and analysed using the FlowJo software (V10, Tree Star, Ashland, OR, USA).

### Statistical analysis

For *P* value calculations, *χ*^2^*-*analysis was used for analysing discrete numbers; Student’s *t*-test (two-tailed) was used for analysing distributed numbers; one-way analysis of variance was used for the multiple comparison of wound-healing experiments. A difference was considered significant when *P* value was less than or equal to 0.05. Error bars in the graphs represent mean±s.e.m. Asterisks indicate a significant difference between two groups. n.s. stands for not significant. Each experiment presented was repeated at least twice, with replicates showing consistent statistic significance.
