# Supplementary figures and images for: Extracellular HSP60 triggers tissue regeneration and wound healing by regulating inflammation and cell proliferation
Source: NPJ Regen Med. 2016 Oct 27;1:16013–. doi: 10.1038/npjregenmed.2016.13 (PMC5605149; doi:10.1038/npjregenmed.2016.13)

Suppl. Fig. 1

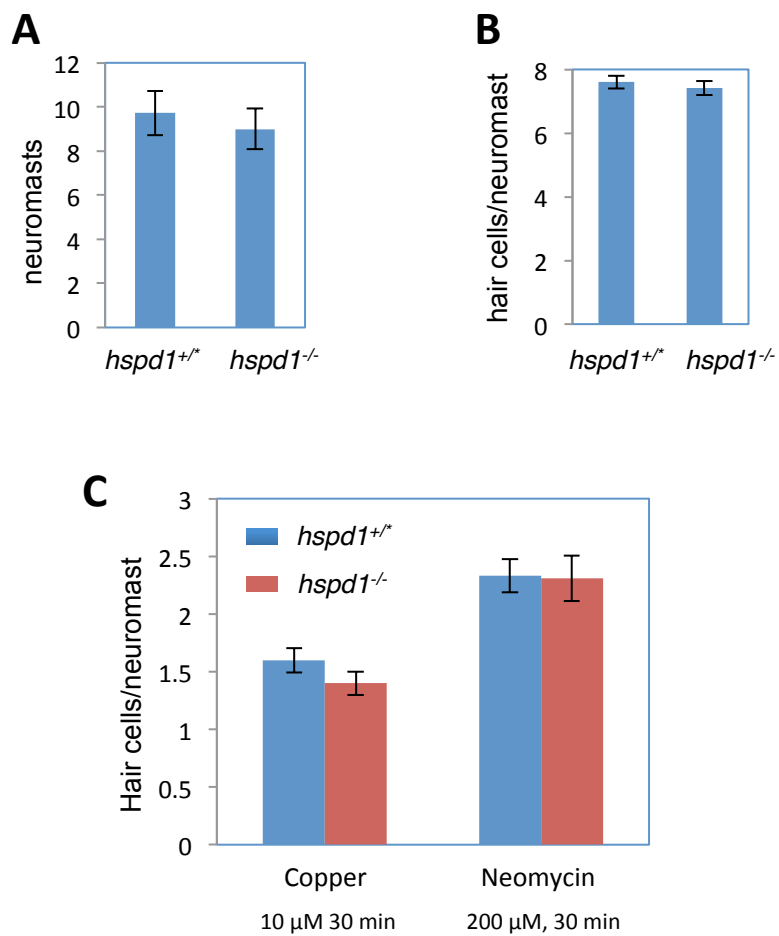

Suppl. Fig. 2

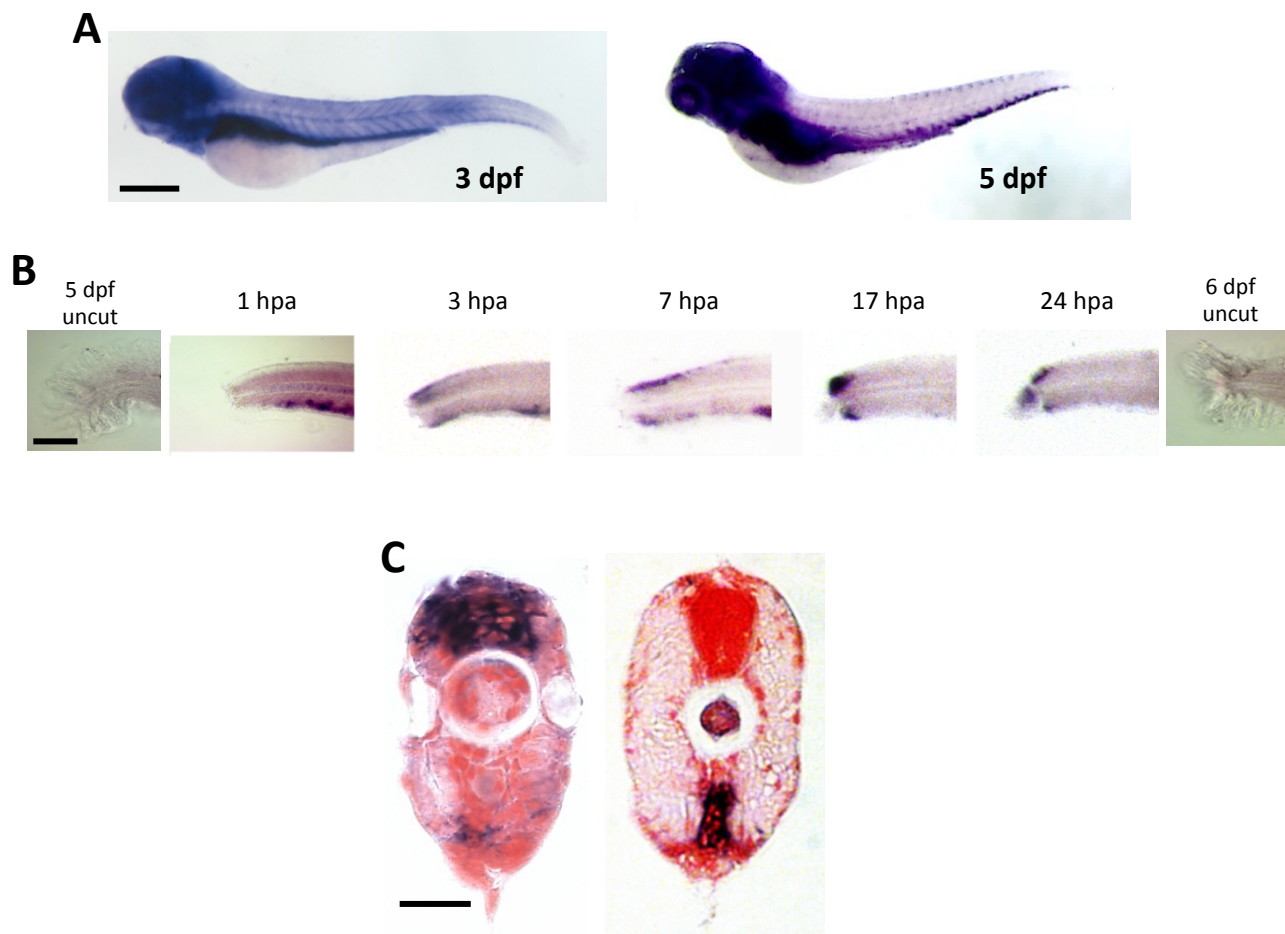

Suppl. Fig. 3

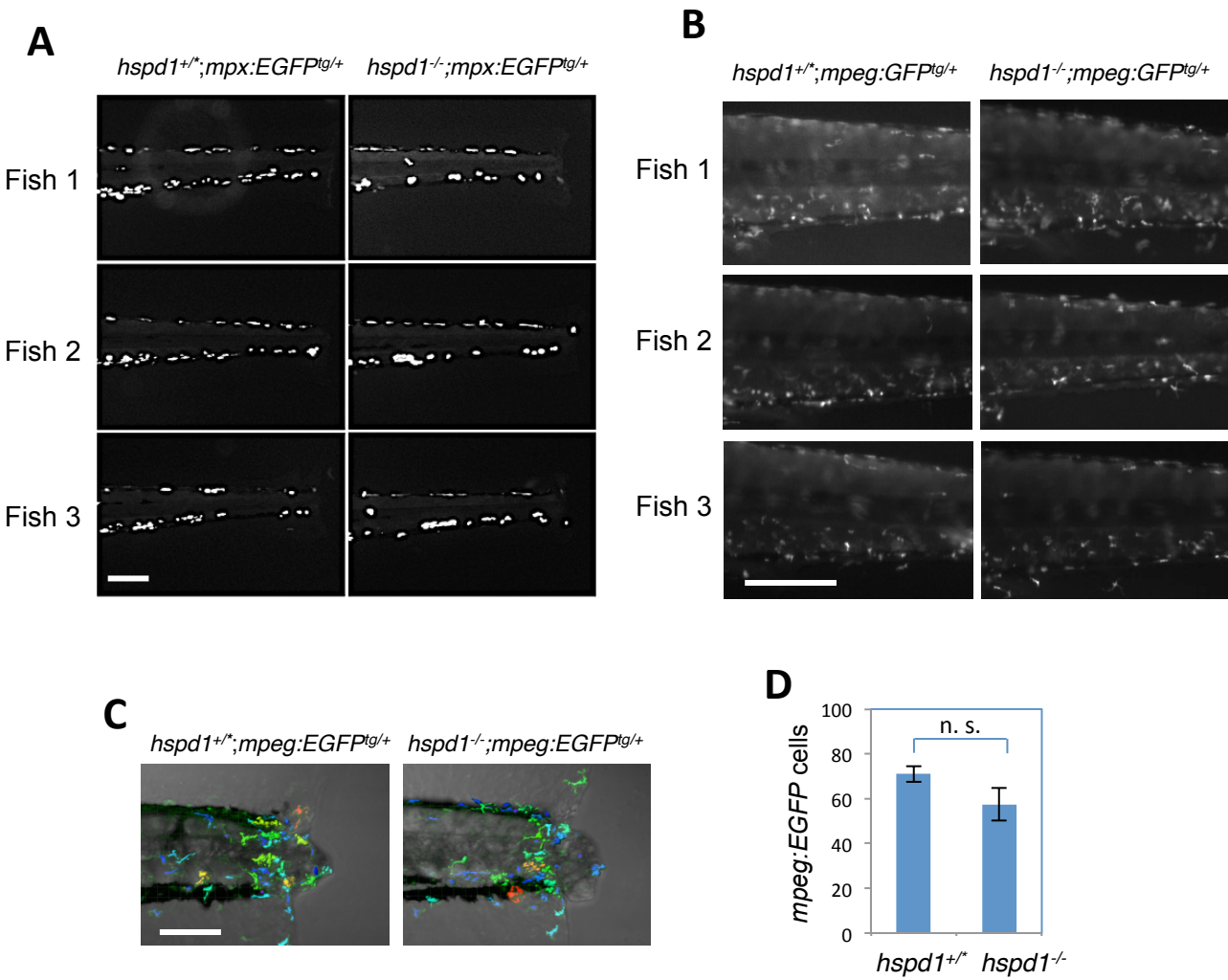

Suppl. Fig. 4

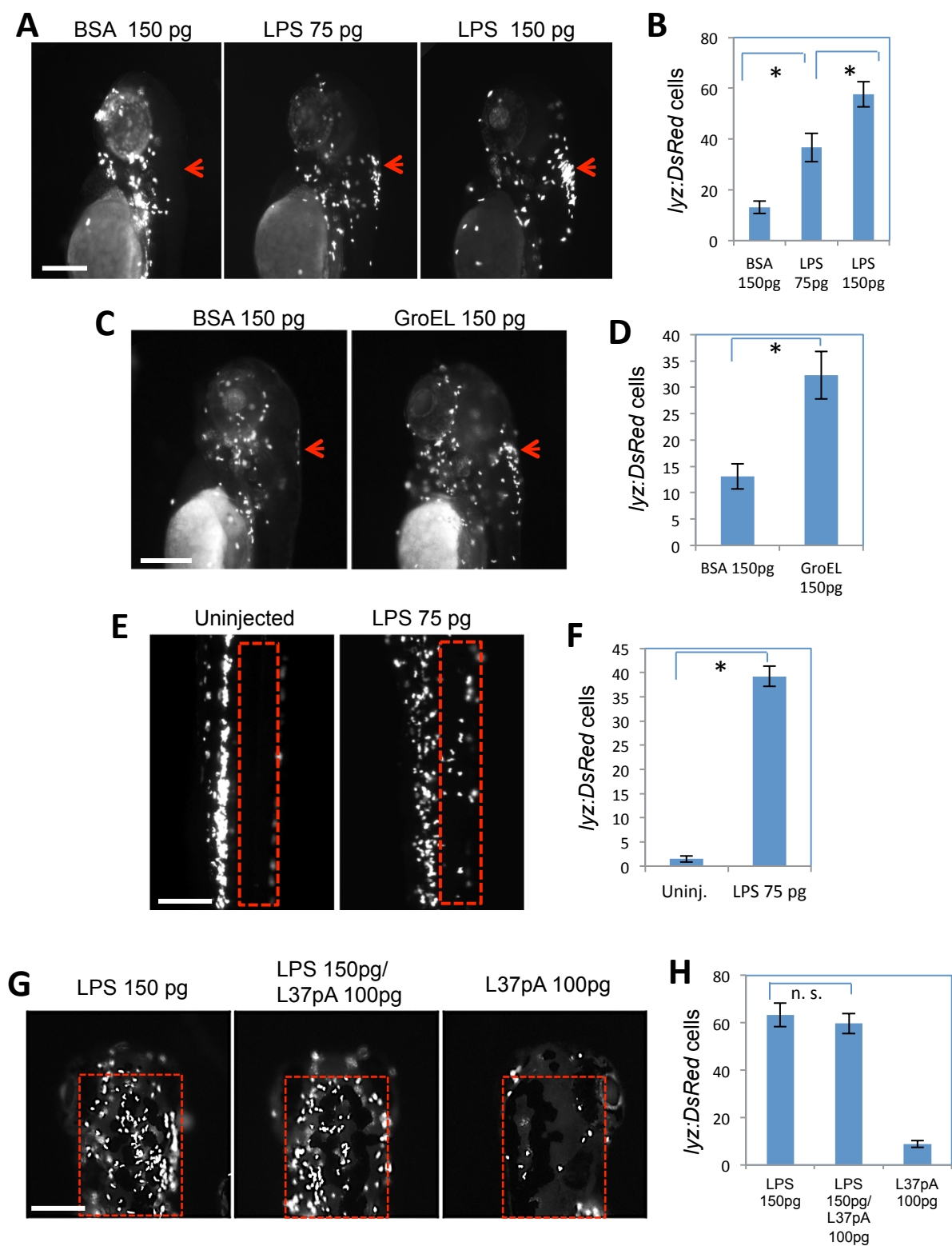

Suppl. Fig. 5

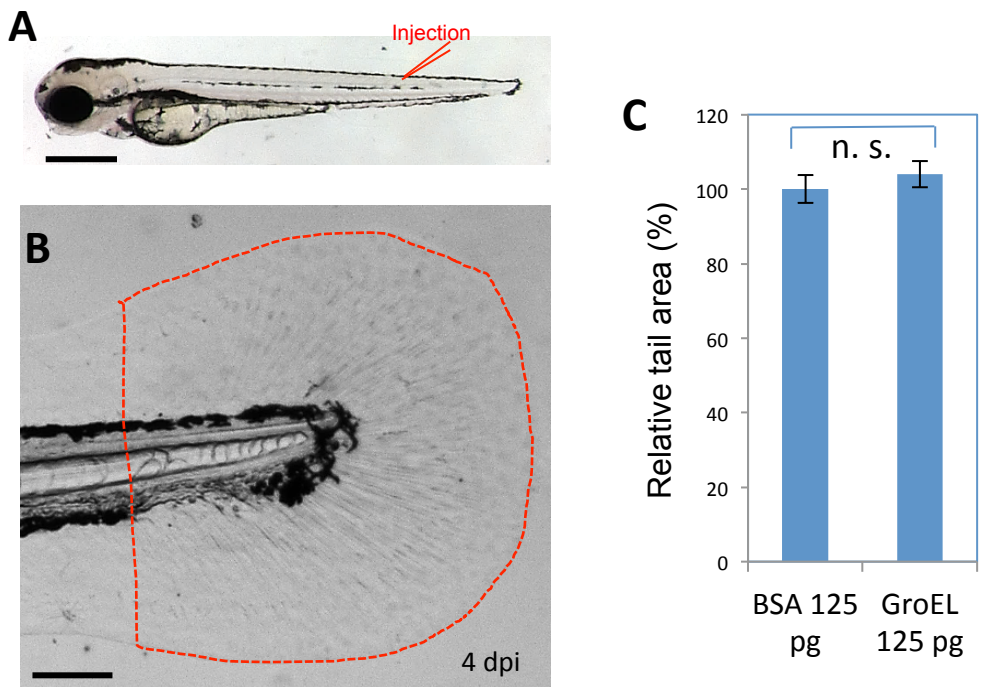

Suppl. Fig. 6

**A**

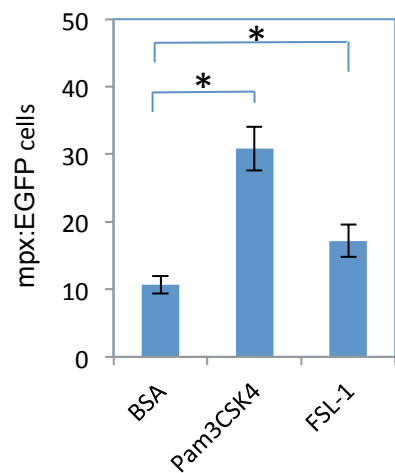

**B**

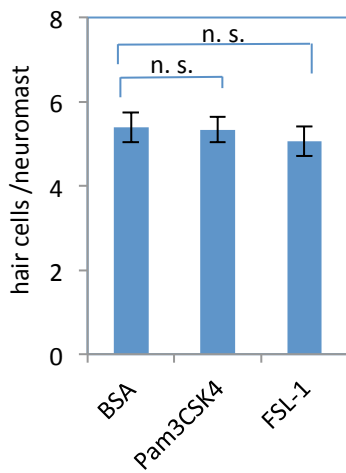

Suppl. Fig. 7

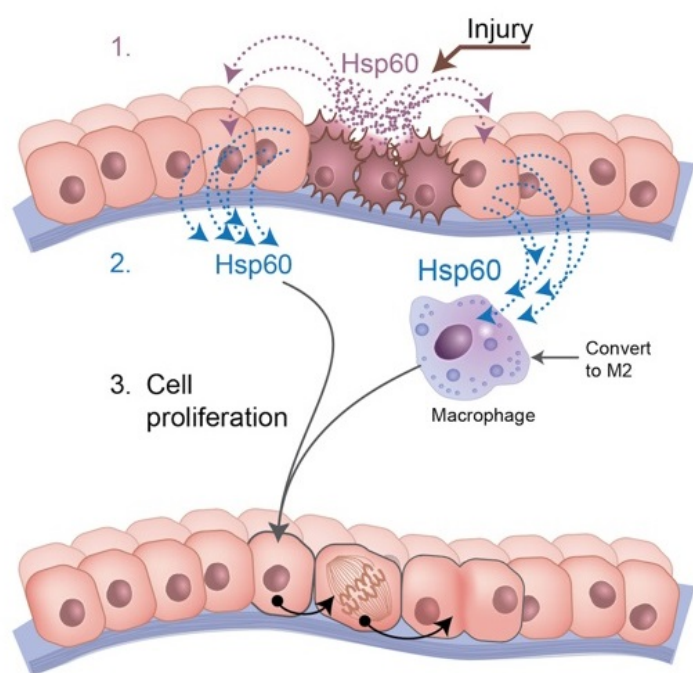

Supplement: Supplementary Figures [file npjregenmed201613-s1.pdf]
